# Relevance of the world health organization in a multipolar world in solving global health challenges

**DOI:** 10.3389/fpubh.2022.1037734

**Published:** 2022-11-10

**Authors:** Ranjit Kumar Dehury

**Affiliations:** School of Management Studies, University of Hyderabad, Hyderabad, India

**Keywords:** criticism of WHO, health diplomacy, World Health Assembly (WHA), International Health Regulation (IHR), epidemic, health emergencies

## Abstract

There have been many criticisms about the World Health Organization (WHO) in the last decade. In a multipolar world, there are rivalries between nations and geopolitical regions. However, health issues remain outside the murky world of politics due to their far-reaching consequences on human society. The power conferred on the WHO is very significant in protecting the health and well-being of the global population. As a neutral organization, the WHO is supposed to uphold people's rights to health, especially in controlling diseases of international importance. The paper highlighted the significant roles of the WHO in leadership issues, research and development, solving disputes among countries, providing resources for low-performing regions, regulating international health laws, responding to a humanitarian crisis, and communicating during the crisis. Further, evidence from global literature critically analyzed the enforcement role of WHO on international health regulations (IHRs).

## Introduction

With constant upheaval of world power centers, there are challenges in global health. At the dawn of the twenty-first century, the world seems to be in a multipolar state with a large political economy of nations. The world is not under one superpower, which would make it unipolar. With emerging economies like India, China, South Africa, Brazil, and many European countries, the world is multipolar now. Even African and South-East Asian countries are also emerging in economic power and diplomatic negotiations for health-related concerns. No more is the world lobbying around the United States of America (USA) and Russia, making a cold war of the past. The developments in the health sector follow global geopolitics, which ultimately decides global health diplomacy. Therefore, international global bodies must address health issues according to the need of space and time. As far as healthy human life is concerned, all the regions of the world are equally important. Other socio-economic and political issues should not infringe on healthcare governance globally.

Earlier, the WHO provided central leadership in the health sector for global health (1). The WHO has faced challenges at various stages in the scientific development and administration of healthcare (2). With a tradition of non-partition and an independent nature, the WHO should have high morals and sanctity. Usually, irrespective of providing funding and other tangible resources, no country puts pressure on the decision-making process of the WHO on health administration anywhere across the world (1). In other words, no one can put pressure to take undue advantage of the WHO's functions. In the past, the WHO had a commendable influence in war-torn territories, humanitarian crises, and epidemics (1). The work of the WHO has been notable in biomedical research and collaboration concerned with solving global medical crises. The WHO is well known for providing help with the global burden of disease on every continent.

Unlike many United Nations bodies, which acted partisanly and had terrible reputations, the WHO earned a fair amount of prestige (1). Despite not being generously funded, in the past the WHO was able to do its jobs with efficiency. Many countries found it great to work with WHO despite not being a member of the United Nations Organization (UNO). Many countries have membership in the WHO without participating in the larger geopolitical groups and the United Nations.

Any international organization not performing its desired role would be irrelevant in the crucible of time. There may be challenges in doing the job right, but it is necessary to remain relevant by accomplishing the job nonetheless. Criticism and overwhelming internal weakness make an organization inefficient. Hence, fresh notions and hard-hitting ideas are required in the WHO to make it more relevant in the coming decades (2). The world will face more challenges due to the movement of people, resources, and political thoughts in the coming days. Therefore, exemplary work by an international organization would help solve problems with proactive steps.

The world is like a household when it comes to many health emergencies. If one member suffers from ill health, others will be affected to some extent, whether physically, mentally, or spiritually. Restoring harmony with minimal damage without loss of time is a challenge. Without functional organizations in the health sector, there may be severe challenges in the global community. Hence, stewardship is required to restore the situation. Similarly, the macro parameters of health care achievement and management of health crises are the job of WHO (3). The regulations of WHO empower to enact sooner to quell the health crisis. Further, WHO and its multiple arms in research and development, program implementation, and resource mobilization must be effective. There is no excuse to avoid the crisis and keep blaming others. It is high time for the WHO to progress in multiple ways.

At the same time, beneficiaries, nation-states, multilateral agencies, and humanitarian groups must be active in helping out in the crisis to restore health. Overall, the restoration of health in human society is not just limited to the direct beneficiaries but also the whole ecosystem. The systems approach with emerging concepts like “one health” needs immediate attention without losing time. The scientific community is already upbeat with evidence from concepts like “one health” in society. This evidence must be translated into policies with progress in the health of the nations.

Authors argue that despite the global health crisis, the WHO faces daunting challenges in violating International Health Regulations and payed a minimal role in response to COVID-19 concerns (4). Recommendations converge toward cooperation and mutual strategic trust for overall progress in the health sector. Under the umbrella of the WHO, there is a requirement to work toward common interest, convergent operations on development, and resource allocation to combat COVID-19. Further, the WHO has to accelerate the mission of health diplomacy to reduce inequality by simplifying access to diagnostics, therapeutics, and vaccines, considering them as a global public good (4).

The paper focused on themes like leadership in research and development, role in solving disputes among countries, provision of resources for low-performing regions, regulating international health laws, response to the humanitarian crisis, and communication at the time of crisis for understanding the details of functions of the WHO. All the thematic areas are discussed critically with available evidence from the literature. The paper consulted search sites like PubMed, Scopus, Web of Science, and JSTOR to unravel the issues of critical concerns of WHO.

## Leadership in development and research

The WHO, since its inception, projected to take leadership in health and development in the world arena. Technical assistance on health is the job of the WHO, irrespective of the level of development of a country. So that there would be a free flow of information, improving public health and timely intervention in the world. Many epidemics need immediate intervention, such as Ebola and COVID-19 in recent times. However, the WHO also depends on various other countries for proper investigation, which delays the matter to a great extent. Overall, the lack of leadership in investigating diseases and outbreaks is detrimental to the health of a large population. International bodies also reiterate the leadership of the WHO in improving access to health for global citizens.

The Oslo Ministerial Declaration in 2007 affirmed that health diplomacy must be part of central foreign policy (5). The ministerial group observed that health security and access to health by the people of the world have far-reaching positive externalities for the development of the world. Hence, the body also suggests measures for access to medicine by making flexibile the Trade-Related Aspects of the Intellectual Property Rights (TRIPS) agreement of the Doha round of Intellectual Property Rights. It has been found that in one geography, everything may not be available, which necessitates the cooperation of the world for the production and distribution of healthcare products. Health is a much-neglected concern in a world where life is precious. A country with a compromised health system cannot ensure stability and security (5).

Authors argue that the coronavirus takes advantage of a divided political structure and non-cooperation (6). The factors necessary besides a strong health structure are social justice in societies, unity at the national level, and global solidarity to fight out a pandemic of the most significant scale. These are things the WHO must focus on to create more value. The current world needs extraordinary coordination across regional and political groups, along with solid relationships among scientists, policymakers, and civil societies. There is a need to take advantage of Global Health Diplomacy (GHD), Vaccine Diplomacy (VD), and Scientific Diplomacy (SD) to usher in a new era of healthcare dynamics for economic development, global health security, just society, and equitable healthcare (6).

## Role in solving disputes among countries

Many disputes originated from the life sciences industries of various countries. Moreover, pharmaceutical issues are also critical to tackling at various forums to solve the issues. The neoliberal policies of global institutions make things more challenging to access medicines in the global south. There is also a requirement for international arbiters to solve issues of public health importance. The WHO aims to solve international health and human services disputes at the international level. As the WHO has expertise in health, it is supposed to advise and recommend measures for the solving of worldwide disputes on health issues. However, due to political influence, there is a lack of proactive steps from the WHO to solve many issues. If one problem arises, the WHO has to send its team to assess the situation. Transboundary laws should be in place to solve the major issues at the global level with dedicated resources. The diplomatic role of the WHO must effectively solve the issues with utmost care. However, in recent decades, the WHO has failed to resolve significant health issues among countries.

Reports confirmed that the US President took steps to cut ties with all WHO activities due to partisan politics. This is evident from the alignment of the WHO with China on the issue of the origin of COVID-19. In contrast, the Chinese president reiterated that China did nothing wrong in its virus notification, which affected the world. Further, the Chinese president announced financial grants to the WHO of $2 billion over 2 years (7). The issues of COVID-19 remain unsolved, with many countries affected by this.

Taiwan was denied membership in the WHO due to inhibitory policy from China. The Chinese government protested against Taiwan's rights, denying the right to be a member. There are also issues in many places affected by diseases and internal health laws in Asia and Africa, which remain unsolved by the WHO regarding access to medicine, healthcare, and Intellectual Property Rights. The disputes between many groups and countries are detrimental to the health and well-being of people.

Scholars argue that the WHO has to create an environment such that there should not be verbal attacks by one country on the other; instead, there must be cooperation to overcome crises (4). A dire crisis in international cooperation would result in failing the patients in terms of receiving essential medications and deceiving front-line healthcare workers. Hence, opinions converge on creating a shared resource pool and allocation (4). The dispute resolution wing must come into action to usher in global cooperation worldwide.

## Providing resources for low-performing regions

The WHO has a fair amount of resources to implement various plans and programs relating to the health of underdeveloped countries. A dedicated part of the resources used to contain pandemics in developing countries is also available. However, the resources often do not reach the target audience to develop health and overall well-being. The danger of an epidemic does not just threaten the local population but also affects neighboring countries, and even sometimes those far off. The low-income regions of the world face difficulty controlling communicable and lifestyle diseases. Resource-starved countries need a lot of funding and technical support to fight these diseases. The WHO providing financial and technical support is supposed to provide healthcare services. There was a time when low-income countries needed resources for fighting deadly diseases like HIV/AIDS, malaria, and tuberculosis without sufficient resources and technical knowledge. Further, they do not have laboratories and medicines to fight against these fatal diseases. In global solidarity, the WHO has to take the lead for crowdfunding and provide high-end laboratory support to control diseases and reduce the mortality rate among a large population of developing countries.

The WHO raises funds from member countries and philanthropic organizations to fund the much-needed programs. There is also a need to provide health systems-related economic resources to transform the programs. The WHO needs to improve governance for the participation of developing countries optimally. Hitherto, it has been seen that despite the provision of appropriate resources, controlling diseases was a distant dream in developing countries due to a lack of governance and corruption issues. However, in recent decades WHO also has had a severe lack of financial resources.

Reports found that the WHO is facing a resource crunch. The primary source of funding was formerly the contribution by member countries according to the assessment of contribution by the World Health Assembly of WHO. In the 1970s, around 62% of the budget came from mandatory contributions from member states, which declined to 18% in recent times (4). Even the Director General of the WHO informed the board about the need for increasing contributions. The director believed some funds were allocated after one major global outbreak. However, everything was forgotten afterwards once the epidemic receded. This means nothing less than failing to plan, which leads to planning to fail. The effort of the WHO can only be strengthened by increasing the budget (8, 9).

The African region receives grants in a different form, which are often insufficient to handle a significant outbreak. Moreover, LMIC are deprived of resources to fight many communicable and non-communicable diseases, including significant pandemics; this needs a meticulous approach to bridge the gap.

## Regulating international health laws

There are many international trade and commerce regulators for the smooth functioning of business across the globe. The regulators ensure good practices by consulting various stakeholders for the outcome. Many countries have bilateral and multilateral diplomatic engagements to facilitate business worldwide. The matters of health and development are featured in many treaties and businesses relationship in a highly globalized world. The WHO often devises laws and regulations on healthcare and public health. The WHO acts as an arbitrator in many ways for the progress of international regulation on healthcare. Further, the regulations are democratically aligned with international conferences and conventions, which are agreed upon by member states. The WHO enforces laws and regulations for the welfare of humanity. However, it has been seen that the enforcement of regulations has been hampered over the last decade (2). The factors like political pressure, failure of diplomacy, and corruption pave the way for complacency in the WHO.

The WHO has to be neutral in every way possible to help countries with health emergencies. A neutral body following established procedures and laws is necessary for the development of the world. Without health regulations, there may be a disaster due to epidemics and fatalities worldwide. There are also overlapping trade and commerce laws with health laws. In this case, deciding to improve the population's health is tricky. Many pressure groups and nation-states continuously act as obstacles in various ways to derail the law enforcement power of the WHO.

International health laws are essential for the well-being of humanity. The WHO must be strong enough to enforce these regulations per established policies. In a multipolar world, there are issues and challenges for the WHO. However, with its technical and diplomatic channels, the WHO needs to be impressive enough to bring the importance of international regulations (2).

The WHO succumbs to the pressure of various nations, which leads to securing well-being. It faces the daunting challenge of violations of International Health Regulations (IHR) by many countries. Even, the WHO has not used its authority to investigate epidemics independently to enforce IHR worldwide. However, the recent punitive action by the USA to drastically reduce funds for the WHO will not help solve the enforcement of IHR in the world (10). Evidence found that solidarity among members of the WHO would help enforce international laws. Hence, WHO must be empowered with enforcement plans based on evidence-based and scientifically geared protocols (10, 11).

The IHRs change over time depending on the need of the hour. These are primarily adopted to combat outbreaks at the global level on a large scale. The United Nations ratified a set of iconic IHRs after a decade of severe acute respiratory syndrome (SARS), which is expected to create international coordination during public health emergencies (11). By ratifying the IHRs, one country must notify the WHO about all the public health concerns to form a Public Health Emergency of International Concern (PHEIC) consortium. However, without enforcement agencies, the WHO is toothless if a member country fails to give notice for any reason (3). Whatever the situation regarding the non-compliance of IHRs, there is a severe threat to humanity. Though PHEIC has successfully deployed to control diseases like Polio, H1N1, Ebola, and Zika, in the case of COVID-19, the committee has not done much (11).

In the context of the COVID-19 pandemic, many things went wrong in the decision-making process of the WHO and action was slow (12). International committees found the inefficiency of WHO in declaring PHEIC by almost 5 months, which led to a hefty toll worldwide in terms of mortality and morbidity. The WHO also failed to ensure travel bans and enforcement of IHRs to contain COVID-19 worldwide. Further, the hazy communications of the WHO made the situation critical. There had previously been successful events from the efforts of the WHO to tackle Ebola in 2014, and everything was declared in time (13–15).

There is a requirement for technical assistance, particularly training and follow-up, to improve IHR worldwide (16–18). Understanding the politics of the border movement and following IHR is needed to improve global health. On many occasions the movement of people is an obstacle in following IHR (19). There is a need for substantial and sustained increases in investments by WHO and various countries to prepare for global health emergencies with effective IHR (20–22). There is a need to share administrative powers by the WHO with various actors for effective decision-making and implementation of IHR rather than a top-down approach (23, 24).

## Response to humanitarian crisis

Due to various causes, there have been humanitarian crises worldwide. So, many well-established intergovernmental, government, and non-government organizations take steps to establish order. Humanitarian crises can happen in any part of the world, and they need immediate interventions according to United Nations conventions and other agreements. The WHO is among various organizations supposed to jump to the fore as soon as possible on health issues. The WHO is well accepted in many countries with its humanitarian assistance as a neutral party. Collaborations with various other agencies may bring value to the healthcare system within the WHO. The technical assistance and health services provided by an agency like the WHO are among the most vital (3). Many countries and worldwide forums ignore the vast amount of collateral damage in a humanitarian crisis. However, swift operation by WHO can reduce the concern for the people. However, the WHO is found to be irrelevant in its work in tackling the humanitarian crisis.

## Communicating global emergencies

Communication of health emergencies remains a global challenge in a time of complex public health issues. Therefore, the role of the WHO is very important in communicating messages worldwide (12). There is a requirement of utmost sensibility in communicating scientific facts for the more significant benefit of the nations. Any mistake by the authority may lead to chaos. Further, to contain outbreaks and epidemics, it is necessary to have functional and practical communication media. The current dispensation is not sufficient to control the situations of health emergencies. Instead, there is a requirement of the community and intergovernmental bodies for the effective communication and dissemination of messages. For example, without knowing the details of health emergencies, if it is communicated at the global level, there may be havoc regarding the economy, which is not acceptable by the countries. Instead of doing a good service through health communication, it may escalate the disaster in different forms. Hence, sensible declaration and management of health emergencies are necessary.

The WHO plays a role in assessing and communicating health emergencies on the global stage. There is a need to empower health communication worldwide so far as epidemics are concerned. However, the WHO does not fulfill the job to the fullest extent worldwide (12). Here the countries of the global south face more challenges than the developed world.

## Influence of geopolitics on the WHO

The WHO is an international body that has to take the concerns of the entire world on health and development (3). It has regional offices in all the important geographies to tackle health emergencies and global health security. Overall, there is a firm conviction in the WHO to counter the world politics of health with direct presence and involvement. Though the headquarters are still in Geneva, the WHO can reach any corner of the world without losing much time. However, in recent times, it has been affected by many geopolitical concerns relating to the health politics of the nations. Many powerful countries influence the body to a great extent in different forms. Geopolitical concerns usually affect the decision-making process of the WHO. Not just in deciding the provision of healthcare support but also in influencing the scientific decisions of the WHO. Over decades, science has remained borderless and cultureless, out of politics, but the WHO cannot maintain that neutral role in scientific decisions. There are many accusations against WHO for being partisan globally (2). A partisan nature not just affects the working of WHO but also adds less value to the process of multilateralism.

Global geopolitics is affecting the functioning of the WHO at different levels. This also hampers the resource generation of the WHO. Big philanthropists are losing faith in the WHO and criticizing the entire process the WHO's functioning. There are charges on WHO being run by some groups of countries (2). The recent developments during the COVID-19 pandemic are one of the concerns.

Due to the One China policy respected by many nation-states, Taiwan was kept outside the ambit of WHO activities. The WHO was founded on controlling communicable diseases with non-partisan principles. The WHO must be apolitical to pave the way for creating values worldwide. Chinese pressure holds Taiwan as a ghost island, which is neither part of a nation nor a nation in itself. However, during the pandemic, Taiwan's work was a model for the world to replicate for COVID-19 management (25–27). Authors found that the WHO failed to manage COVID-19 across the world in contrast to the success of Taiwan. The region's geopolitics do not help Taiwan to be included in the WHO despite its effort in meaningful value creation in global health diplomacy (28).

The WHO is not free from the politics of a specific group of nations and their diplomatic approaches. During the pandemic, major trade and cultural exchanges continued. Often, the WHO could not implement regulations due to concerns about the geopolitics of various nations. Overall, general geo-economics and politics significantly influence the functioning of the WHO.

## Negotiating intellectual property for better access to medication

Intellectual property is granted to encourage creativity and innovation. The incentives from Intellectual property are supposed to create more value in the future. The discovery of drugs and vaccines has a positive impact on medical development. The world has suffered from various diseases for some time. Medications are also available to treat many diseases. However, the price of medication becomes so high that it becomes difficult for the ordinary person to access those treatments. Third-world countries face an uphill task of matching the budget of innovators and pharmaceutical companies worldwide. The WHO must bridge the gap by negotiating medication prices for vulnerable sections of society. Even during a pandemic, there is no license waiver for better access and treatment of the world community. The WHO should take the discussion in such a way that there should be easy access to and production of vaccines and medicines worldwide.

The negotiation by WHO must be for the equitable distribution of rare global healthcare resources, like medications and vaccines, during health emergencies. The waiver of patents has helped access to drugs in the past, which the WHO must adopt every time during a global health security crisis. The WHO can negotiate with stakeholders like industry, nations' pressure groups, and multilateral bodies for the efficient production and distribution of medicines.

The role of WHO is very limited in trade negotiations, making the entire world suffer from a shortage of vaccines in the recent past. Despite developing vaccines with public money, licensing vaccines in favor of private firms limits access to the global south. There should be a relaxation of vaccine licensing in which the WHO plays a significant role.

The role of WHO in solving vaccine issues during COVID-19 is not noteworthy. LMIC especially faced many challenges in receiving vaccines timely and equitably (29, 30). In the absence of the intervention of the WHO the wealthier countries took undue advantage of vaccine distribution (31, 32). Hence the role of the WHO in ushering goodwill for vaccine diplomacy is inevitable.

## Conclusion

This article highlights the issues of leadership in research and development, its role in solving disputes among countries, provision of resources for low-performing regions, regulating international health laws, response to the humanitarian crisis, and communication at the time of crisis, which, by and large, comes under the ambit of the WHO at international level. However, the WHO was found to be ineffective in many of the parameters. Various reports and evidence by researchers confirm the feeble nature of the WHO in engendering goodwill. In a multipolar world, the WHO needs to be non-partisan and focus on controlling outbreaks and promoting health and well-being. There is a need for sufficient financial provision and good governance in the leadership of WHO.

To be relevant in the next decade, the WHO must efficiently manage global health security and public health diplomacy to create more value. The objectives of WHO must be redefined to match the aspirations of global citizens, which is essential to make life better across countries. Further, the WHO must tackle the issues of geopolitics and geo-economics aspects to implement various health programs per the scientific requirement for health and well-being.

## Data availability statement

The original contributions presented in the study are included in the article/supplementary material, further inquiries can be directed to the corresponding author.

## Author contributions

The author confirms being the sole contributor of this work and has approved it for publication.

## Funding

This work was supported by the University of Hyderabad [UoH-IoE by MHRD (F11/9/2019-U3 (A))].

## Conflict of interest

The author declares that the research was conducted in the absence of any commercial or financial relationships that could be construed as a potential conflict of interest.

## Publisher's note

All claims expressed in this article are solely those of the authors and do not necessarily represent those of their affiliated organizations, or those of the publisher, the editors and the reviewers. Any product that may be evaluated in this article, or claim that may be made by its manufacturer, is not guaranteed or endorsed by the publisher.

## References

[1] Park K. Park's Textbook of Preventive and Social Medicine. Jabalpur: Banarsidas Bhanot (2019). p. 1–10.

[2] Fidler DP. The World Health Organization Pandemic Politics: The good, the bad, an ugly future for global health. (2020). Available online at: https://www.thinkglobalhealth.org/article/world-health-organization-and-pandemic-politics (accessed May 22, 2022).

[3] Maxmen A. Why did the world's pandemic warning system fail when COVID hit? Nature Springer Science and Business Media LLC. (2021) 589:499–500. 10.1038/d41586-021-00162-4 33500574

[4] Javed S Chattu VK. Strengthening the COVID-19 pandemic response, global leadership, and international cooperation through global health diplomacy. Health Promot Perspect. (2020) 10:300. 10.34172/hpp.2020.48 33312925 PMC7723006

[5] Ministers Of Foreign Affairs Of Brazil France Indonesia Norway Senegal South Africa And Thailand. Oslo Ministerial Declaration—global health: a pressing foreign policy issue of our time. Lancet. (2007) 369:1373–8. 10.1016/S0140-6736(07)60498-X 17448824

[6] AlKhaldi M James N Chattu VK Ahmed S Meghari H Kaiser K. Rethinking and strengthening the Global Health Diplomacy through triangulated nexus between policymakers, scientists and the community in light of COVID-19 global crisis. Glob Health Res Policy. (2021) 6:12. 10.1186/s41256-021-00195-2 33845923 PMC8041471

[7] Associated Press News. China delayed releasing coronavirus info, frustrating WHO. Available online at: https://apnews.com/3c061794970661042b18d5aeaaed9fae (accessed May 22, 2022).

[8] Mahbubani K. The Great Convergence. New York: Public Affairs (2020).

[9] WHO WHO Report of the Director-General 146th 146th Meeting of the Executive Board (2020). Available online at: https://www.who.int/director-general/speeches/detail/report-of-the-director-general-146th-meeting-of-the-executive-board (accessed May 22, 2022).

[10] Venkatapuram S. A WHO fit for the 21st century. ORF (2020). Available online at: https://www.orfonline.org/expert-speak/a-who-fit-for-the-21st-century-67926/ (accessed May 22, 2022).

[11] Gostin LO Habibi R Meier BM. Has global health law risen to meet the COVID-19 challenge? Revisiting the international health regulations to prepare for future threats. SSRN Electronic Journal Elsevier. (2020) 48:376–81. 10.2139/ssrn.3598165 32631189

[12] The The Economics Times (Mar 11 2021). WHO's pandemic response: From criticism to Nobel? 2021. Available online at: https://economictimes.indiatimes.com/news/international/world-news/whos-pandemic-response-from-criticism-tonobel/articleshow/81443977.cms?utm_source=contentofinterest&utm_medium=text&utm_campaign=cppst (accessed May 22, 2022).

[13] Maurice J. Expert panel slams WHO's poor showing against Ebola. Lancet. (2015) 386:e1. 10.1016/S0140-6736(15)61253-3 26183412

[14] World Health Organization. Report of the Ebola Interim Assessment Panel. (2015). Available online at: https://www.who.int/csr/resources/publications/ebola/report-by-panel.pdf?ua=1 (accessed May 22, 2022).

[15] Mackey TK. The ebola outbreak: catalyzing a “shift” in global health governance? BMC Infect Dis. (2016) 16:699. 10.1186/s12879-016-2016-y 27881085 PMC5121963

[16] Razavi A Collins S Wilson A. Comment on Carson et al. strengthening global health security–lessons learned from Public Health England's International Health Regulations strengthening project. Globalization and Health. (2022) 18:50. 10.1186/s12992-022-00844-2 35570295 PMC9107589

[17] Sharma UC Attwood K Pokharel S. Quantitative analysis of international health regulations annual reports to identify global disparities in the preparedness for radiation emergencies. BMJ Open. (2022) 12:e052670. 10.1136/bmjopen-2021-052670 PMC9445785 36691150

[18] Vennis IM Boskovic M Bleijs DA Rutjes SA. Complementarity of International Instruments in the Field of Biosecurity. Front Public Health. (2022) 10:894389. 10.3389/fpubh.2022.894389 35712271 PMC9195852

[19] Piper J Gomis B Lee K. “Guided by science and evidence”? the politics of border management in Canada's response to the COVID-19 pandemic. Front Polit Sci. 2022:17. 10.3389/fpos.2022.834223

[20] Clarke L Patouillard E Mirelman AJ Ho ZJM Edejer TT-T Kandel N. The costs of improving health emergency preparedness: a systematic review and analysis of multi-country studies. eClinicalMedicine. (2022) 44:101269. 10.1016/j.eclinm.2021.101269 35146401 PMC8802087

[21] Burci GL Forman L Hoffman SJ. Introduction to a special issue on reforming the international health regulations. Int Organ Law Rev. (2022) 19:1–10. 10.1163/15723747-19010009

[22] Velásquez G. The World Health Organization Reforms in the Time of COVID-19. Vaccines, Medicines and COVID-19. (2022). p. 93–108. 10.1007/978-3-030-89125-1_6

[23] Vese D. On the administrative powers of the WHO: a lesson from the pandemic. Eur J Health Law. (2022) 1:1–16. 10.1163/15718093-bja10093

[24] Forman L Sekalala S Meier BM. The world health organization, international health regulations and human rights law. Int Organ Law Rev. (2022) 19:37–62. 10.1163/15723747-19010002

[25] American Institute in Taiwan. U.S.-Taiwan joint statement (2020). Available online at: https://www.ait.org.tw/u-s-taiwan-joint-statement/ (accessed May 22, 2022).

[26] Chang JLJ. Taiwan's participation in the world health organization: the US “facilitator” role. In: The Future of United States, China, and Taiwan Relations. New York: Palgrave Macmillan (2010). p. 167–87. 10.1057/9780230118966_9

[27] Chen PK. Universal participation without Taiwan? a study of Taiwan's participation in the global health governance sponsored by the World Health Organization. In: Asia-Pacific Security Challenges. Springer, Cham (2018). p. 263–81.

[28] Yin JD-C. WHO, COVID-19, and Taiwan as the Ghost Island. Global Public Health Informa UK Limited. (2021) 16:1267–82. 10.1080/17441692.2021.1890184 33635180

[29] Gorodensky A Kohler JC. State capture through indemnification demands? Effects on equity in the global distribution of COVID-19 vaccines. J Pharm Policy Pract. (2022) 15:50. 10.1186/s40545-022-00442-y 35986397 PMC9388983

[30] de Bengy Puyvallée A Storeng KT. COVAX, vaccine donations and the politics of global vaccine inequity. Glob Health. (2022) 18:26. 10.1186/s12992-022-00801-z 35248116 PMC8897760

[31] Zhang D Jamali AB. China's “weaponized” vaccine: intertwining between international and domestic politics. East Asia. (2022) 39:279–96. 10.1007/s12140-021-09382-x 35079216 PMC8776365

[32] Peng Y. Politics of COVID-19 vaccine mandates: Left/right-wing authoritarianism, social dominance orientation, and libertarianism. Pers Individ Dif. (2022) 194:111661. 10.1016/j.paid.2022.111661 35431382 PMC8995254

